# The fundamentals of fetal alcohol spectrum disorder: Evaluation of an awareness‐raising webinar

**DOI:** 10.1111/acer.70251

**Published:** 2026-02-19

**Authors:** Stewart McDougall, Ruth Brown, Jen Shields, Sarah Brown, Rachel Burn, Suzanne O'Rourke

**Affiliations:** ^1^ The Fetal Alcohol Advisory Support and Training Team, Health in Social Science, College of Arts, Humanities, and Social Science University of Edinburgh UK; ^2^ NHS Ayrshire and Arran UK

**Keywords:** fetal alcohol Spectrum disorder, prenatal alcohol use, training program

## Abstract

**Background:**

Health professionals in the UK and internationally often lack knowledge of prenatal alcohol exposure (PAE) and fetal alcohol spectrum disorder (FASD). Raising awareness of PAE and FASD across health and social care sectors is vital to support Scotland's implementation of neurodevelopmental pathways. This study evaluated whether engaging in the *Fundamentals of FASD* contributed to change in (i) attitudes toward the health advice given to pregnant women, (ii) attitudes toward PAE, (iii) attitudes toward FASD, and (iv) knowledge of FASD. Furthermore, this study examined whether knowledge and attitudinal changes are maintained to 12 months posttraining.

**Methods:**

A total of 1327 attendees attended across 14 workshops. Of these, 1005 completed an initial evaluation questionnaire assessing their attitudes and knowledge toward PAE and FASD (pretraining [“T1”]). Repeated‐measure follow‐up responses were collected immediately after training (“T2”; *n* = 525); at 3 months (“T3”; *n* = 128); and at 12 months (“T4”; *n* = 157).

**Results:**

Linear mixed models revealed trainees demonstrated significant improvement on all measures at posttraining but demonstrated a varied pattern at the T3 and T4 follow‐up. Attitudes toward the health advice about alcohol use in pregnancy and alcohol use in pregnancy generally demonstrated no change between T2 and T4, indicating improvement was sustained from T2 to T4. In contrast, attitudes toward FASD and FASD knowledge were observed to significantly decline from T2 through to T4, albeit remaining higher than the pretraining.

**Conclusions:**

Attending the *Fundamentals of FASD* was associated with significant improvement in knowledge and attitudes toward PAE and FASD. A decline in FASD knowledge across time, albeit remaining above pretraining scores suggests ongoing or refresher training is required. Provision of training within continuing professional development frameworks may help sustain awareness and encourage healthcare professionals to be better equipped in supporting individuals affected by PAE and/or FASD.

## Graphical abstract

This study examined the effectiveness of an online training session, the *Fundamentals of Fetal Alcohol Spectrum Disorder (FASD) for Health and Social Care Professionals,* which was effective in changing knowledge of and attitudes toward prenatal alcohol exposure and FASD. The training session was associated with increased knowledge and more appropriate attitudes immediately post training. Improvements attenuated but remained above pretraining levels at 12 months. Such training represents a pragmatic and scalable component of a broader systems approach to improving FASD knowledge.

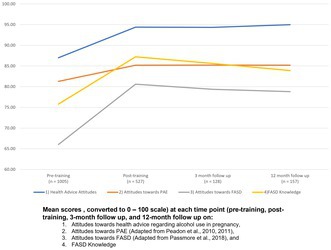

## INTRODUCTION

Fetal alcohol spectrum disorder (FASD) is a critical yet often overlooked public health challenge globally. Characterized by a range of physical, cognitive, and behavioral impairments resulting from prenatal alcohol exposure (PAE), FASD can have profound impacts on affected individuals' quality of life and functioning (Scottish Intercollegiate Guidelines Network (SIGN), 2019). The prevalence of FASD is estimated at 1.17% globally, with between 3% and 5% of the UK population predicted to have FASD (Lange et al., 2017; McCarthy et al., 2021; Popova et al., 2017).

### Training and awareness of FASD among health and social care professionals

Limited training on and knowledge of the symptoms, diagnosis, and management of FASD are reported among health, education, justice, and social work professionals (McCormack et al., 2022). Rates of training among professionals are variable; a UK study reported that just over half of pediatricians (54.8%) and just over one in five midwives (21.3%) had received training on FASD (Howlett et al., 2019). Similarly, 35% of healthcare professionals reported limited knowledge of alcohol use in pregnancy guidelines (Mukherjee et al., 2015), and one in five midwives were citing outdated guidance (Smith et al., 2021). Knowledge of FASD prevalence was low, with only 19.8% of midwives correctly reporting national estimates (Howlett et al., 2019). Limited knowledge of alcohol guidelines and FASD contributes toward a lack of confidence in assessing and diagnosing FASD, leading to underdiagnosis and inadequate support for affected individuals (McCormack et al., 2022). Professionals consistently report a strong desire for more comprehensive training and educational resources related to FASD (Smith et al., 2021), with growing calls for such UK‐specific training to be developed (Howlett et al., 2019; National FASD, 2022). Equipping professionals with accurate knowledge of FASD is vital to ensuring individuals and families receive appropriate and effective support (Coons et al., 2016, 2018; Reid et al., 2022). Furthermore, professionals' beliefs that FASD is stigmatizing (McCormack et al., 2022), and beliefs about the “prognosis” following a diagnosis of FASD may serve as barriers, contributing to a lack of diagnosis (Choate & Badry, 2019). Qualitative research suggests caregivers disagree with this sentiment, instead seeking a diagnosis to better understand and explain their child's needs (Domeij et al., 2018). These findings highlight the need for training to address misinformation and misconceptions related to FASD (McCormack et al., 2022) and alcohol use during pregnancy (Oni et al., 2019) so that individuals with FASD may be better supported.

While there are numerous examples of training developed for professionals about FASD (see Bagley et al. (2023) for a scoping review of available e‐learning modules), there are few studies that have examined changes in knowledge, attitudes, or practices following training. One example—the *Reframe the Behavior* program developed for youth justice workers—led to significant improvements in knowledge and informed attitudes toward FASD, alongside positive practice changes in supporting individuals with neurodevelopmental (ND) challenges (Passmore et al., 2021). Similarly, Australian healthcare workers who attended FASD‐specific training were more likely to contribute to the diagnosis of FASD and ask about alcohol during pregnancy posttraining (Reid et al., 2020). These studies suggest that training is an effective intervention to improve knowledge, attitudes, and practice among professionals.

Nonetheless, two key limitations of the past training programs are of note. Previous training programs typically provided attendees with an overview of FASD (e.g., its clinical presentation). However, the social factors that can contribute to the lived experience of FASD, the diagnosis and management of FASD, and the societal and self‐stigma faced by those affected by FASD and PAE is typically not incorporated into training content (Bagley et al., 2023). Furthermore, there has been little evaluation of the existing resources to determine their effectiveness in improving professional knowledge and practice. With some exceptions demonstrating changes in knowledge and attitudes posttraining (e.g., Passmore et al., 2021), few studies have administered pre‐ and posttraining measurements of knowledge to measure change in attendees' knowledge and attitudes. This lack of rigorous assessment means that the true impact of these resources remains largely unknown. Moreover, no studies have assessed whether these changes in knowledge or attitudes over longer periods determine whether changes in knowledge and attitudes are maintained long‐term.

### Scottish context

FASD is under‐recognized and under‐diagnosed in many countries. It is estimated that a 67‐fold increase in diagnostic capacity is required to meet the need in Canada (Popova et al., 2024). Similarly, the Scottish Parliament notes that as many as 99% of FASD cases may be unrecognized (Scottish Parliament, 2021). Taken together with the findings from the evaluation of a pilot specialist diagnostic clinic, which found that the demand for FASD assessments would outstrip the capacity of a national clinic (McGruer & Shields, 2018), Scotland has devolved responsibility to regional services through ND pathways (Maciver et al., 2023; Rutherford et al., 2021). The ND pathway approach is a whole‐system, multidisciplinary model that organizes assessment and intervention around a child or young person's functional profile and context, rather than single condition diagnostic silos. Aligned with the Getting It Right For Every Child (GIRFEC) framework (Scottish Government, 2008, 2022), the National Neurodevelopmental Specification for Children and Young People (Scottish Government, 2021) prioritizes early identification, proportionate supports, and access to help without a formal diagnosis. The National Neurodevelopmental Specification for Children and Young People (Scottish Government, 2021) advocates for the inclusion of FASD within local ND pathways. Successfully embedding FASD into ND pathways, and the recognitive and effective differential and comorbid diagnosis, requires significant numbers of staff to be trained to support equity of access across a wide geographical region. *The Fundamentals of FASD for Health and Social Care Professionals* (hereafter the “Fundamentals of FASD”) sought to address the “awareness” level of training in Scotland's FASD Training and Competency Framework (Shields et al., 2025).

Enhancing professionals' knowledge of PAE and FASD in support of the ND pathway approach is key for several reasons. The importance of teratogenic exposures, including PAE, is reflected in their incorporation into clinical assessment and triaging tools in pediatric and ND pathways to support formulation (Rutherford et al., 2024). This in turn creates a need for healthcare professionals to understand the importance of enquiring about alcohol use in pregnancy in a consistent, unbiased, supportive, and nonstigmatizing manner. Crucially, PAE is associated with the comorbid diagnosis of other neurodevelopment conditions, particularly attention deficit hyperactivity disorder (ADHD; Chasnoff et al., 2015; Clark et al., 2024). The importance of differentiating idiopathic ADHD and ADHD in the context of PAE is highlighted by the different responsiveness to stimulant medications, owing to different underlying mechanisms (Ritfeld et al., 2022). Currently, there are low rates of diagnosis of FASD and low rates of inclusion of FASD in ND pathways across Scotland (Maciver et al., 2025). Enhancing knowledge of PAE and FASD is key in enabling appropriate diagnosis and care across the ND pathway (Olson et al., 2023).

This study evaluates a novel targeted training program, designed to enhance the knowledge of and informed attitudes toward FASD and PAE among health and social care workers. Specifically, the current study aimed to examine changes in knowledge and attitudes toward PAE and FASD following attendance at *The Fundamentals of FASD* webinar, and to examine the stability in these changes over time. The study has two key aims. Firstly, to evaluate whether engaging in the *Fundamentals of FASD* contributed to change in (i) attitudes toward the health advice given to pregnant women, (ii) attitudes toward PAE, (iii) attitudes toward FASD, and (iv) knowledge of FASD. Secondly, to examine whether any knowledge and attitudinal changes are maintained up to 12 months posttraining.

## METHOD

### The Fundamentals of FASD

The *Fundamentals of FASD* is an online, 90‐min training session, consisting of approximately 75 min of delivered content and approximately 15 min for questions from attendees. The training adopted a didactic approach, with opportunities for interaction limited to questions via the webinar platform chat function, which, time permitting, were addressed as posted or at the end of the session. The *Fundamentals of FASD* training was developed by the FAAST Team, based on training delivered alongside a pilot diagnostic clinic (McGruer & Shields, 2018). Furthermore, the training sought to address the learning objectives shown in Table 1, reflecting the first level (informed; awareness) of the FASD Training and Competency Framework (Shields et al., 2025). The session covered two main components: (1) Alcohol use and prenatal alcohol use; covering culture and contributors to alcohol use, the prevalence of PAE in the UK, and the factors associated with PAE, and (2) FASD covering prevalence, clinical presentation, and the importance of early identification and support. The training was tailored to a Scottish and UK audience, incorporating UK statistics and definitions for standard drinks, low risk drink guidelines, and binge drinking definitions. The training was delivered 14 times between November 2022 and July 2024. Each session was delivered by two members of the FAAST Team, with each training led by a clinical psychologist or a pediatrician, both with significant expertise in FASD, accompanied by either the training officer or a postdoctoral research fellow.

**TABLE 1:** Learning outcomes for *The Fundamentals of FASD* training.

	Learning outcome
1	Have an increased understanding of alcohol use in Scotland
2	Have an increased understanding of alcohol units and alcohol guidelines
3	Have an increased understanding of the potential factors that can contribute toward alcohol use during pregnancy
4	Have an increased understanding of how alcohol can affect the developing fetus
5	Have an increased understanding of fetal alcohol spectrum disorder (FASD) and how it can affect people
6	Be aware of the prevalence of FASD
7	Be aware of sources of further information and signposting opportunities

*Note*: Learning outcomes reflect the competency statements of the “Informed, Awareness” level of the FASD Training and Competency Framework (Shields et al., 2025).

### Procedure

The training was advertised through NHS Scotland and social care mailing lists and via social media. While the training was funded by the Scottish Government and tailored to the Scottish context, registrations outside of Scotland were accepted. Participants registered to attend via an Eventbrite page. Instructions on accessing the webinar and an invitation to complete the pretraining questionnaire were then sent to all registrants. The questionnaire opened with an information and consent sheet, and participants provided consent before starting the survey.

The questionnaire comprised demographic questions and four outcome measures: (1) attitudes toward the health advice regarding alcohol use in pregnancy; (2) attitudes toward PAE; (3) attitudes toward FASD; and (4) an FASD knowledge measure. Participants were invited to complete the measures at four time points: prior to engaging with the training (“T1”), directly after the training (“T2”), at a 3‐month follow‐up (“T3”), and at a 12‐month follow‐up (“T4”).

Participants received the invitation to the first questionnaire upon registration. Immediately after the training session, registrants who had completed the pretraining questionnaire were sent the posttraining questionnaire. At each data collection wave, participants received two reminders, each 1 week apart, to complete the questionnaire. Participants were offered a certificate of completion for attending the training and completing the T1 and T2 questionnaires. This study was approved by the University of Edinburgh Health in Social Sciences Ethics Committee (CLPS208).

### Measures

Participants completed the same four measures across all time points. Additionally, at the posttraining data collection, participants completed four items asking them to rate their perception of the training and an open‐text question for suggestions for improving the training.

#### Attitudes toward health advice

A three‐item measure was designed by the research team to capture attitudes toward Scotland's Chief Medical Officer health advice regarding alcohol use in pregnancy (e.g., “Women should avoid alcohol when trying to become pregnant”). Participants were asked to rate their agreement on a five‐point Likert scale where 1 = Strongly Disagree and 5 = Strongly Agree. Scores ranged from 3 to 15, with higher scores indicative of more informed attitudes toward health advice. The current study found this measure displayed good internal consistency at each time point (*α* = 0.850–0.948).

#### Attitudes toward PAE

The “Attitudes toward PAE” scale (Peadon et al., 2010, 2011) was used to measure participants attitudes toward alcohol use during pregnancy. Participants were asked to rate their agreement to statements assessing their attitudes toward PAE (e.g., “Drinking alcohol can lead to life‐long disabilities in a child”) on a five‐point Likert scale where 1 = Strongly Disagree and 5 = Strongly Agree. Scores ranged from 12 to 60, and higher scores indicated more informed attitudes toward alcohol use during pregnancy. In the current study, the scale demonstrated adequate internal consistency at each timepoint (*α* = 0.710–0.824), whereas previous research reported a lower internal consistency (*α* = 0.628; Keating et al., 2025).

#### Attitudes toward FASD

The “Attitudes toward FASD” scale was adapted from the scale developed by Passmore et al. (2018) to measure attitudes toward FASD among justice professionals. As the original scale was developed for justice professional in Australia, several adaptations were made to the original measure. First, the two items, “FASD occurs primarily in Aboriginal families,” and, “FASD is relevant to the justice system” were removed to allow the measure to be less culturally‐ and justice‐specific. A further two justice‐specific items were re‐worded (e.g., “I am familiar with how FASD might affect young people and adults involved in the criminal justice system” was re‐worded as “I am familiar with how FASD might affect people's lives”). Three items reflecting beliefs about stigma or stigmatizing beliefs were added by the research team; “The emphasis on FASD is stigmatising to women,” “Most birth mothers who drink when pregnant know it can harm the baby,” and “The benefits of a diagnosis of FASD do not outweigh the harm it can cause to families.” The resultant scale contained 16 items scored on a five‐point Likert scale where 1 = Strongly Disagree and 5 = Strongly Agree. Scores ranged from 16 to 80. Higher scores were indicative of more informed attitudes toward FASD. While used in previous studies (Passmore et al., 2018, 2021), the psychometric properties of these measures were not reported but were demonstrated to be amenable to change through training programs (Passmore et al., 2021). The current study found this measure displayed good internal consistency at each time point (*α* = 0.784–0.897).

#### FASD knowledge

To measure knowledge of key facts about FASD, the research team developed a 14‐item measure containing a mixture of multiple choice and true/false questions to measure the participants factual knowledge about FASD (e.g., “True or false, the early and accurate diagnosis of FASD reduces the risk of negative long‐term outcomes”). Knowledge was summed to a total score where questions were scored 1 for a correct response, and 0 for an incorrect response. Total possible scores ranged between 0 and 14. Questions asking participants to select all options (e.g., “What are some of the reasons that individuals with FASD may go undiagnosed or receive an incorrect diagnosis?”) were scored by dividing the 1 score available by the number of correct options. Therefore, partial scores were possible.

### Data preparation

Data were collected via Qualtrics. When data collection was complete, data were downloaded and imported into IBM SPSS for preparation and analysis. Data preparation involved the removal of incomplete responses (i.e., responses that did not provide a complete response to at least one of the outcome measures) and identification of duplicate responses. Duplicate responses occurred when participants signed up for multiple sessions and thus received multiple invites to complete the survey. Duplicate responses were identified by the provided email addresses. Reasons for multiple sign‐ups were not sought from participants but may include being unable to attend the original session and signing up for a subsequent session. Multiple patterns of responses were identified among participants with multiple registrations. If participants were identified to have completed multiple responses at a wave of data collection, only the earliest response was retained. This was particularly relevant at T1 to ensure that participants had not previously completed the *Fundamentals of FASD* training.

### Data analysis

Prior to the main analysis, attrition rates were calculated between the T1, T2, T3, and T4 samples and participant demographics across the four samples were compared using chi‐squared tests. Scores were then calculated for each of the four knowledge and attitude measures (i.e., attitudes toward health advice, attitudes toward PAE, attitudes toward FASD, and knowledge of FASD) at each of the four time points. To evaluate the change and stability of knowledge and attitude score improvement posttraining, four separate linear mixed models (LMM) were then fitted onto the four outcome measures (i.e., attitudes toward PAE), across the four time points (T1 to T4). This method is recommended over other longitudinal analysis techniques (e.g., repeated‐measure ANOVAs) given LMMs ability to accommodate missing‐at‐random data points; thus improving statistical power (Krueger & Tian, 2004). As LMMs assume the residuals, not the raw data, are normally distributed (Schielzeth et al., 2020), Q‐Q plots of the residuals were used to assess the normality of residual distributions (see Figure S1). Participant identification number was included as a random intercept in the models to account for between‐subjects variance in pretraining attitudes and knowledge toward FASD and PAE. Fixed effects included time of measurement (T1, T2, T3, and T4) and participant's previous training experience (experience/no experience). To evaluate whether scores significantly improved between T1 and T2 scores; and scores did not significantly change between the T2, T3, and T4 measurements, post hoc pairwise comparisons of estimated marginal means (EMMs), with Bonferroni correction, were conducted. All models were estimated with restricted estimated maximum likelihood with Satterthwaite approximation. Data analysis was conducted on SPSS version 29. QRS Nvivo was used for the management and analysis of the qualitative comments. Content analysis was used to identify common themes among comments.

## RESULTS

### Participant demographics

Registrations across the 14 training sessions totaled 2218, of whom, 1327 (59.8%) attended a training session. Of the attendees, 1072 (80.8%) commenced the pretraining questionnaire. Usable (i.e., consented to participation, and completed the demographic questionnaire and at least one outcome measure) responses were received from 1005 responses (93.8%) at T1. Of these, usable responses (i.e., at least one outcome measure completed) were received from 521 responses at T2, from 128 at T3, and 157 at T4.

As shown in Table 2, at T1, participants were predominantly Scottish (*n* = 933, 92.8%) and while the training was specific to Scotland, 6.2% of participants were from the other nations of the UK (e.g., England and Northern Ireland) or international. Nursing (28.4%), social care/social work (27.3%) and allied health (16.3%) were the most represented professional groups. Participants had, on average, worked in their current role for 11 years. Less than half of the participants (41.6%) reported having received some training about FASD or PAE, or experience of working with individuals with FASD, prior engaging in the *Fundamentals of FASD*. Attendance at presentations (23.6%); professional experience working with individuals with FASD (22.7%); or other CPD opportunities (18.7%) were the most commonly reported prior training experiences. Fewer participants reported receiving training as part of their undergraduate (9%), or postgraduate study (9%). Table 2 shows the demographic characteristics of the sample at each time point.

**TABLE 2:** Demographic characteristics across waves (T1–T4).

	Present at each data collection wave:							
	T1 (*n* = 1005)		T2 (*n* = 527)		T3 (*n* = 128)		T4 (*n* = 157)	
	*n*	%	*n*	%	*n*	%	*n*	%
Professional background								
Psychology/Mental health	119	11.8%	68	12.9%	20	15.6%	28	17.8%
Social work/Social care	274	27.3%	137	26.0%	31	24.2%	27	17.2%
Nursing and midwifery	285	28.4%	136	25.8%	19	14.8%	37	23.6%
Medics	32	3.2%	20	3.8%	5	3.9%	9	5.7%
Allied health	164	16.3%	93	17.6%	34	26.6%	36	22.9%
Education	71	7.1%	36	6.8%	10	7.8%	11	7.0%
Other	60	6.0%	37	7.0%	9	7.0%	9	5.7%
Prior training								
Yes	418	41.6%	236	44.8%	65	50.8%	78	49.7%
No	587	58.4%	291	55.2%	63	49.2%	79	50.3%
Country								
Scotland	933	92.8%	496	94.1%	119	93.0%	146	93.0%
UK	58	5.8%	27	5.1%	7	5.5%	10	6.4%
International	4	0.4%	2	0.4%	1	0.8%	0	0.0%
Missing	10	1.0%	2	0.4%	1	0.8%	1	0.6%
Years of experience								
Less than 5	326	32.4%	171	32.4%	41	32.0%	50	31.8%
5–10	231	23.0%	111	21.1%	28	21.9%	32	20.4%
11–15	116	11.5%	57	10.8%	12	9.4%	13	8.3%
16–25	178	17.7%	107	20.3%	27	21.1%	34	21.7%
More than 25	103	10.2%	52	9.9%	17	13.3%	22	14.0%
Missing	51	5.1%	29	5.5%	3	2.3%	6	3.8%

Patterns of attrition are further analyzed in Table 3. Participants were assigned to mutually exclusive groups based on their engagement. There was a significant association between prior training and attrition category, albeit with a small effect size (*χ* ^2^(4, *N* = 1005) = 9.923, *p* = 0.042, Cramer's *V* = 0.099). Post hoc cell wise tests using adjusted standardized residuals (ASR) indicated that those without prior training on FASD were marginally more likely than expected to drop out after T1 (ASR = 2.0) compared to those with prior training (ASR = −2.0). Similarly, there was a significant association, again with a small effect size, between professional background and attrition categories (*χ* ^2^(24, *N* = 1005) = 40.53, *p* = 0.019, Cramer's *V* = 0.10). Post hoc cell wise tests using ASR indicated that allied health professionals were over‐represented among those completing all waves of data collection (ASR = 3.4), while nursing and midwifery were under‐represented (ASR = −3.0).

**TABLE 3:** Demographics by drop out categories.

	Dropped out:								Intermittent (*n* = 119)		*Χ* ^2^	Df	*p*
	Complete (*n* = 51)		After T1 (*n* = 436)		After T2 (*n* = 341)		After T3 (*n* = 58)						
	*n*	%	*n*	%	*n*	%	*n*	%					
Professional background											40.53	24	**0.019**
Psychology/Mental health	9	17.6%	46	10.6%	35	10.3%	9	15.5%	20	16.8%			
Social work/Social care	7	13.7%	125	28.7%	100	29.3%	16	27.6%	26	21.8%			
Nursing and midwifery	5	9.8%	137	31.4%	97	28.4%	13	22.4%	33	27.7%			
Medics	4	7.8%	11	2.5%	11	3.2%	1	1.7%	5	4.2%			
Allied health	17	33.3%	64	14.7%	49	14.4%	10	17.2%	24	20.2%			
Education	5	9.8%	31	7.1%	25	7.3%	4	6.9%	6	5.0%			
Other	4	7.8%	22	5.0%	24	7.0%	5	8.6%	5	4.2%			
Prior training											9.92	4	**0**.**042**
Yes	27	52.9%	166	38.1%	138	40.5%	31	53.4%	56	47.1%			
No	24	47.1%	270	61.9%	203	59.5%	27	46.6%	63	52.9%			
Country^a^											3.21	4	0.523
Scotland	47	92.2%	398	91.3%	322	94.4%	54	93.1%	112	94.1%			
UK	4	7.8%	29	6.7%	16	4.7%	3	5.2%	6	5.0%			
International	0	0.0%	2	0.5%	1	0.3%	1	1.7%	0	0.0%			
Missing	0	0.0%	7	1.6%	2	0.6%	0	0.0%	1	0.8%			
Years of experience											22.00	20	0.341
Less than 5	15	29.4%	140	32.1%	111	32.6%	19	32.8%	41	34.5%			
5–10	9	17.6%	109	25.0%	72	21.1%	14	24.1%	27	22.7%			
11–15	3	5.9%	58	13.3%	36	10.6%	8	13.8%	11	9.2%			
16–25	16	31.4%	66	15.1%	67	19.6%	9	15.5%	20	16.8%			
More than 25	8	15.7%	44	10.1%	31	9.1%	6	10.3%	14	11.8%			
Missing	0	0.0%	19	4.4%	24	7.0%	2	3.4%	6	5.0%			

*Note*: T1 = pretraining score; T2 = posttraining score; T3 = 3‐month follow‐up scores; T4 = 12‐month follow‐up scores. Bold values indicates significant *p* values.

^a^ Categories for “Country” collapsed to “Scotland” and “All Other” for chi‐squared analysis due to violation of assumptions.

### Change in knowledge and attitudes

A series of four LMMs were conducted to evaluate change in scores across the four training outcomes, from T1 to T4, correcting for participants' previous training or professional experience with FASD prior to engaging with the *Fundamentals of FASD*. A full overview of the results across the four models is presented in Table 4.

**TABLE 4:** Results from linear mixed model (LMM) analyses across the four attitudes and knowledge scores.

Effects	Model 1 (AIC = 8207.99)				Model 2 (AIC = 11198.69)				Model 3 (AIC = 11843.36)				Model 4 (AIC = 6008.97)			
	(i) Attitudes toward Health Advice				(ii) Attitudes toward PAE				(iii) Attitudes toward FASD				(iv) FASD Knowledge			
	*B*	SE	*t*	*p*	*B*	SE	*t*	*p*	*B*	SE	*t*	*p*	*B*	SE	*t*	*p*
Fixed																
Intercept (T4 reference)	16.39	0.593	27.65	**0.001**	54.47	1.70	31.97	**0.001**	85.83	1.89	45.45	**0.001**	13.99	0.360	38.86	**0.001**
T1 vs. T4	−0.825	0.193	−4.28	**0.001**	−1.61	0.468	−3.45	**0.001**	−7.32	0.551	−13.28	**0.001**	−0.989	0.101	−9.79	**0.001**
T2 vs. T4	0.039	0.202	0.191	0.893	0.123	0.488	0.253	0.801	1.69	0.576	2.94	**0.003**	0.560	0.105	5.30	**0.001**
T3 vs. T4	−0.051	0.263	−0.193	0.857	0.090	0.635	0.141	0.888	0.469	0.754	0.622	0.534	0.232	0.137	1.69	0.091
Training	0.464	0.125	−3.72	**0.001**	0.399	0.361	−1.10	0.269	4.42	0.399	−11.07	**0.001**	0.522	0.076	−6.84	**0.001**

	B	SE	ICC		B	SE	ICC		B	SE	ICC		B	SE	ICC	
Random																
Residual	4.43	0.223			23.27	1.21			34.18	1.61			1.12	0.058		
Between‐subject	1.17	0.218	0.209		16.44	1.62	0.414		17.38	1.78	0.337		0.705	0.075	0.385	

*Note*: T1 = pretraining score; T2 = posttraining score; T3 = 3‐month follow‐up scores; T4 = 12‐month follow‐up scores. Reference category for time contrasts is T4. All models estimated with restricted estimated maximum likelihood with Satterhwaite approximation. Bold values indicates significant *p* values.

Abbreviations: AIC, Akaike's Information Criterion; B, estimate; FASD, fetal alcohol spectrum disorder; ICC, intraclass coefficient; PAE, prenatal alcohol exposure.

Table 5 shows the Bonferroni‐corrected pairwise comparisons across the four outcome measures for each timepoint. Results for each outcome are discussed in turn below.

**TABLE 5:** Bonferroni‐correct pairwise comparisons across (i) Health advice, (ii) PAE attitudes, (iii) FASD attitudes, and (iv) FASD knowledge scores, across the four time points.

Time of measurement (EMM)	T2 (SE)	T3 (SE)	T4 (SE)
(i) Model 1: Attitudes toward health advice			
T1 (13.45)	0.864*** (0.117)	0.774*** (0.210)	0.825*** (0.193)
T2 (14.31)	/	−0.090 (0.219)	−0.039 (0.202)
T3 (14.22)		/	−0.051 (0.263)
T4 (14.28)			/
(ii) Model 2: Attitudes toward PAE			
T1 (51.03)	1.74*** (0.282)	1.71** (0.515)	1.61** (0.468)
T2 (52.77)	/	−0.034 (0.532)	−0.123 (0.488)
T3 (52.74)		/	−0.090 (0.635)
T4 (52.66)			/
(iii) Model 3: Attitudes toward FASD			
T1 (58.37)	9.01*** (0.336)	7.79*** (0.612)	7.32*** (0.551)
T2 (67.38)	/	−1.22 (0.504)	−1.69* (0.486)
T3 (66.16)		/	−0.469 (0.754)
T4 (65.69)			/
(iv) Model 4: FASD Knowledge			
T1 (10.62)	1.55*** (0.061)	1.22*** (0.111)	0.989*** (0.101)
T2 (12.17)	/	−0.327* (0.115)	−0.560*** (0.105)
T3 (11.85)		/	−0.232 (0.137)
T4 (11.61)			/

*Note*: T1 = pretraining; T2 = posttraining; T3 = 3‐month follow‐up; T4 = 12‐month follow‐up. *p < 0.05, ***p < 0.001.

Abbreviations: EMM, estimated marginal mean; SE, standard error.

#### Attitudes toward health advice

Model 1 found significant main fixed effects of time of measurement [*F*(3, 1195.39) = 21.80, *p* < 0.001] and previous training or professional experience [*F*(1, 793.27) = 13.88, *p* < 0.001] on change in Attitudes toward Health Advice scores. Bonferroni‐corrected pairwise comparisons of the EMMs revealed a significant improvement in Attitudes toward Health Advice scores from T1 to T2, T3, and T4 (all *p* < 0.001), whereas T2 to T4 scores did not significantly differ (all *p* = 1.00), implying stability of score improvement from T2 onward. The effect of previous training or experience suggests that participants showed improvements, regardless of prior training or experience. The random intercept variance (1.17; SE = 0.220, ICC = 0.209) suggested moderate between‐subject variability in pretraining Attitudes toward Health Advice scores.

#### Attitudes toward PAE

Model 2 found a main fixed effect of time of measurement [*F*(3, 994.98) = 15.17, *p* < 0.001], but not of previous training or professional experience [*F*(1, 875.43) = 1.22, *p* = 0.269] on Attitudes toward PAE scores. Bonferroni‐corrected pairwise comparisons of the EMMs revealed a significant improvement in scores from T1 to T2, T3, and T4 (all comparisons *p* < 0.001), with no other comparisons identified as significant, suggesting sustained improvement in PAE attitudes. The random intercept variance (16.44; SE = 1.62, ICC = 0.414) suggested high between‐subject variability in pretraining Attitudes toward PAE scores.

#### Attitudes toward FASD

Model 3 found a main fixed effect of time of measurement [*F*(3, 1198.22) = 271.51, *p* < 0.001] and previous training or professional experience [*F*(1, 993.62) = 122.48, *p* < 0.001] on change in Attitudes toward FASD scores. Bonferroni‐corrected pairwise comparisons of the EMMs revealed a significant improvement in FASD Attitude scores from baseline measurement to all other timepoints (all comparisons *p* < 0.001), with a significant decrease in scores between T2 and T4 (*p* = 0.020). No other comparisons were identified as significant. The random intercept variance (17.38; SE = 1.78, ICC = 0.337) suggested high between‐subject variability in pretraining Attitudes toward FASD scores.

#### FASD knowledge

Model 4 found a main fixed effect of time of measurement [*F*(3, 1013.07) = 231.19, *p* < 0.001] and previous training or professional experience [*F*(1, 840.07) = 46.82, *p* < 0.001] on FASD Knowledge scores. Bonferroni‐corrected pairwise comparisons of EMMs revealed a significant improvement in baseline FASD knowledge to all other times of measurement (all comparisons *p* < 0.001), with a significant decrease in scores from T2 to T3 (*p* < 0.026) and T2 to T4 (*p* < 0.001). The random intercept variance (0.705; SE = 0.075, ICC = 0.385) suggested high between‐subject variability in pretraining FASD Knowledge scores.

### Acceptability of the training, and changes to practice, and suggestions for adaptation to the training

As shown in Table 6, the overwhelming majority of attendees agreed that the training was interesting (95.5%), that the training was useful for their work (95.9%), and that they would recommend the training to others who may be working with individuals with FASD, or women of child‐bearing age (98.1%).

**TABLE 6:** Proportion of participants endorsing items on the acceptability of the *Fundamentals of FASD* training (T2, *n* = 515–517).

Statement	Agreed	Neither	Disagreed
The training kept my interest^a^	95.5%	2.9%	1.6%
I would recommend this training to others who may work with women of child‐bearing age, or who may work with affected individuals^a^	98.1%	0.8%	1.2%
Through the training I became aware of how I can apply what I have learned^b^	89.8%	7.9%	2.3%
I consider what I learnt through the training as useful for carrying out my work^b^	95.9%	2.9%	1.2%

*Note*: Agreed includes both strongly agree and agree, and disagreed included both strongly disagree and disagree.

^a^ *n* = 515.

^b^ *n* = 517.

#### Practice changes

At the T3 and T4 follow‐up, 37.5% (*n* = 48/128) and 45.9% (*n* = 72/157) of participants, respectively, indicated that they had worked with an individual diagnosed with, or suspected to have, FASD or their families, since the training. Participants indicating they had worked with someone with FASD were asked what, if any, changes to their practice the training had supported (T3 = 43 comments, T4 = 62 comments). Participants frequently made positive comments on the training, but omitted information on practice change (i.e., “very informative” [T3 = 42%, T4 = 67%]). Most commonly (T3: 33%, T4: 21%), participants reported the *Fundamentals of FASD* training led to greater consideration of PAE/FASD in their formulation and differential diagnosis. Furthermore, the training was considered a useful stepping stone to seek out additional training. Several participants (T3: 11.6%, T4: 8.1%) indicated that they were sharing the knowledge gained through the *Fundamentals of FASD* with colleagues. A smaller proportion of participants noted that the training was helpful in identifying supports for individuals with FASD, or in developing practice in asking questions about PAE (T3: 11.6%, T4: 6.4%). Barriers to practice change included limited access to diagnostic services, FASD not being considered within services, or difficulties in gaining developmental histories to inform diagnostic assessment, as illustrated by one occupational therapist, who commented:

We have a proportion of our population [Secure Children's Homes] that have neurodevelopmental needs and other diagnoses (ADHD, Autism) [that] do not feel quite right. Now that I have an increased understanding of FASD I feel a little more able to consider this as a possibility. We do struggle to get developmental histories for our patients so this is a barrier to diagnosis.

#### Adaptations to the training

Participants were given an open‐text question at T2 asking “what can we do to improve the FASD training course?” In total 182 (34.7% of the T2 responses) comments were received. Of these, 56 (29.2%) were general positive comments, without substantive suggestions or calls to expand the training and increase the availability. Participants also commented requesting copies of handouts or slides (*n* = 25, 13.7%; “It would be helpful to have some or all of the slides or similar to use for reference after the training”) or on accessibility and access to the webinar platform (*n =* 13, 7.1%; e.g., “Please use less medical jargon, we are not all health professionals”).

Suggestions for additions to the content were made in 53 (29.1%) responses. Participants, while highlighting the value of the training, suggested including additional content on several topics, such as a greater focus on intervention or support, particularly within education, greater inclusion of lived experience, or a greater focus on the differential diagnosis of FASD, ADHD, and autism, as highlighted by one participant:

I would be interested in learning a bit more about the distinction between how FASD presents and ASD and ADHD. I wonder about the number of people out there who have perhaps been incorrectly diagnosed. Equally, I would be interested to think more and hear discussion about co‐morbidity, and potential links between FASD and ASD/ADHD.

Suggestions for changes to the delivery of the training were made by 34 participants. Participants were divided on whether in‐person or online deliver was preferred, acknowledging the trade‐off between increased engagement and the broader reach of online programs. There were also several calls for incorporating more interactive elements into the predominantly didactic format, as well as increasing opportunities for participant discussion. For example, one participant noted: “It would be good if it could have been more interaction [sic], but I appreciate that this is challenging online and with a timescale.”

## DISCUSSION

This study sought to examine the change in knowledge and attitudes toward PAE and FASD in health and social care professionals, following attendance at the *Fundamentals of FASD* awareness‐raising webinar. Moreover, the study investigated whether such changes were sustained over a 12‐month period.

Significant improvement in both knowledge and attitudes toward FASD was observed posttraining. There was, however, a decrease in scores on both measures between posttraining and the 12‐month follow. The 12‐month scores albeit remained above the pretraining score, suggesting that a modest increase is maintained to the 12‐month follow‐up. On the one hand, it is reasonable to posit that attitudes, once altered, can be passively retained over time. On the other hand, sustaining knowledge improvement requires active reinforcement, such as recall or application. Thus, it is likely that while the training changed less‐informed attitudes toward the condition, recall of information was less durable subsequent to a single training session.

This study found that attending *Fundamentals of FASD* improved attitudes toward the provision of health advice about alcohol use during pregnancy, and more informed attitudes about alcohol use in pregnancy. Attitudes were significantly higher at posttraining, indicating that the training was influential on attendees' initially held beliefs. Furthermore, this change was maintained beyond the training, with no significant differences observed between posttraining and 12‐month follow‐up. It should be noted that trainees largely reported appropriate attitudes prior to engaging with the training. Nevertheless, significant change was observed posttraining; and such changes to attitudes did not diminish over time.

Taken together, it appears that the *Fundamentals of FASD* training had meaningful and lasting influence on the trainees' knowledge and attitudes toward FASD and PAE. Such findings suggest training had both influenced the opinions of those who held less‐informed attitudes toward alcohol use during pregnancy; and reinforced understanding of the risks of PAE in those who initially held more informed attitudes. However, the significant decrease in FASD knowledge over time, highlights the need for repeated training, or “refresher” courses, to sustain such initial knowledge improvement. In support of ND pathway approaches, FASD awareness should be universal.

Consistent with previous research both in the UK and internationally, professionals reported limited prior training on FASD (McCormack et al., 2022). The lack of training is recognized as a barrier to the assessment and diagnosis of FASD (Kerimofski et al., 2024). In the current study, few participants identified their qualifying studies or formal training as the source of FASD training, suggesting that health professionals may be entering the workforce without sufficient awareness of FASD. Similarly, research with Australian psychologists highlights a lack of training on FASD in undergraduate and postgraduate studies for psychologists and allied health professionals (Kerimofski et al., 2025). Furthermore, New Zealand health professions reported training on FASD most frequently came from their own research, CPD, or via expert colleagues (Chu et al., 2024; Kerimofski et al., 2025). Given the high prevalence of alcohol use in pregnancy (Popova et al., 2017), and of FASD in the general population (3%–5%; Lange et al., 2017), this highlights a need for ensuring sufficient coverage during the professional training of many health professionals to support equity of access to diagnostic and support services. Furthermore, given the voluntary nature of training offerings like the *Fundamentals of FASD*, those who are already aware of PAE/FASD (even without formal training) may be more likely to engage in such training opportunities, while those who are less aware of the importance of upskilling in FASD may be less likely to attend. Mandating training on FASD for relevant professions (Popova et al., 2020), or ensuring that FASD is covered in qualifying studies (Kerimofski et al., 2024, 2025), may be important avenues to ensure professionals are sufficiently knowledgeable and confident to support individuals with FASD. Participants' responses to the open‐ended questions highlighted some barriers to the implementation of new knowledge to support adequate service provision to individuals with FASD. For example, participants highlighted the challenges in accessing diagnostic services, similar to that noted internationally (Popova et al., 2024). Furthermore, lack of information regarding PAE is an ever‐present barrier to the consideration of FASD in diagnostic formulation (Bakhireva et al., 2018).

Many attendees did not report working with individuals with FASD at the 3‐ or 12‐ months follow‐up. Given FASD's prevalence in the general population, this may indicate that FASD is under‐recognized, indeed the Scottish Government estimates that as many as 99% of FASD cases may be undiagnosed (Scottish Parliament, 2021). The underdiagnosis and under‐recognition of FASD may contribute to a cycle wherein, despite the high prevalence of FASD, professionals encounter few diagnosed cases, leading training engagement, and therefore subsequent recognition of cases, to be limited. In this context, attendee's reports of limited posttraining contact with individual's with FASD further underlines the importance of ensuring that professionals are knowledgeable of the risks of PAE, and services are alert for, and responsive to, potential PAE as per the “informed” level of awareness of the Training and Competency framework (Shields et al., 2025).

### Limitations

There are several limitations that need to be acknowledged in this study. Firstly, there was no comparison group, limiting the interpretability of reported improvement effects. Furthermore, while measures were predominantly adapted from previous studies, ambiguous wording of some items and their applicability for repeated administration were identified. Future research could consider further psychometric validation of measures intended to assess the attitudes toward and knowledge of PAE and FASD. Particularly, given the changes to the “Attitudes toward FASD” scale the construct validity and measurement invariance over time has not yet been established and future research should consider validation of this, and the other measures, in the study.

Furthermore, health professionals who are already interested in PAE or FASD may be more likely to self‐select to attend a voluntary training session, such as the *Fundamentals of FASD*. The advertisements for the study were supported by the Scottish Government and disseminated through the NHS to ensure a wide reach, in an attempt to minimize this potential self‐selection bias. Furthermore, future training initiatives could be embedded into curricula, mandated training, or embedded into the training calendars of professional societies.

There was considerable attrition across the study despite regular reminders being sent to participants at each data collection point. Certificates of completion were issued to participants who completed the pre‐ and postmeasures, but there was no incentive to complete the subsequent waves, which may contribute to the attrition at 3 and 12 months. Analysis of attrition categories revealed significant, albeit small associations between professional background and prior training, such that allied health professionals were over‐represented among those completing all waves of data collection. Furthermore, prior training was associated with completing the T2 follow‐up questionnaire. Such patterns may bias the sample toward those who are more favorably inclined toward FASD or positive bias about the training experience.

While a key strength of this study was the multiple waves of follow‐up, only a small proportion of participants provided data for all four waves. Consequently, LMMs were selected for use, given they are robust against unbalanced repeated‐measure data. Unlike more traditional longitudinal analysis methods (e.g., repeated‐measure ANOVAs) (Krueger & Tian, 2004), LMMs do not require complete observations across each wave of data collection. Instead, missing observations are addressed via restricted maximum likelihood, increasing statistical power and mitigating potential biases. Thus, LMMs are considered appropriate analyses to model score change over time, even when significant attrition is observed (Stolz et al., 2018).

### Future research directions

Further examination and validation of the psychometric properties of the measures used in this study would be beneficial. Future training evaluations could assess additional concepts, including confidence/self‐efficacy to support individuals with FASD, consider the role of moderating variables (e.g., previous training and experience), and include greater examination of stigmatizing beliefs and attitudes about PAE and FASD. The qualitative comments from trainees suggests that the training may have contributed to practice change. Future research could examine posttraining implementation of FASD‐informed practice, and what barriers and facilitators professionals experience when incorporating their new knowledge into practice.

Further consideration of the “active ingredients” of the training offered may be beneficial. Research suggests online CPD training for health professionals is as effective as in‐person (Pagnucci et al., 2023); however, attendees frequently expressed a desire for in‐person training and greater interactivity. Future training evaluations could consider whether in‐person training yields improvements (including in attendance rates) compared with web‐based training packages.

The *Fundamentals* training was funded by the Scottish Government and primarily advertised in Scotland. While the training was open to individuals from outside of Scotland, there was a limited response from the other countries of the UK (e.g., England, Wales, and Northern Ireland). Given the promising results of this study and the limited knowledge of FASD reported in studies of predominantly English healthcare professionals (Howlett et al., 2019; Mukherjee et al., 2015), similar training could be rolled out to health and social care professionals across the UK.

## CONCLUSIONS

This evaluation of the *Fundamentals of FASD for Health and Social Care Professionals* webinar indicates that brief, targeted training can produce meaningful and enduring improvements in the knowledge of and attitudes toward FASD and PAE. Although FASD attitudes and knowledge improved posttraining, these measures attenuated over time, albeit remaining above pretraining scores. This underscores the need for reinforcement through refresher modules, opportunities for application, and integration into routine practice. Universal, curriculum‐embedded FASD training and proactive outreach to under‐served professions or regions may be warranted, given the limited prior training on FASD in prequalification education and to overcome self‐selection into voluntary training. The *Fundamentals of FASD* training represents a pragmatic and scalable component of a broader systems approach, coupled with ongoing learning and service improvements, which may advance consistent, FASD‐aware practice across health and social care.

## FUNDING INFORMATION

The Fetal Alcohol Advisory Support and Training Team received funding from the Scottish Government to deliver the *Fundamentals of FASD for Health and Social Care Professionals* training and to undertake the evaluation of this training. Furthermore, the FAAST Team receive funding from the Scottish Government to provide related training and consultancy.

## CONFLICT OF INTEREST STATEMENT

The FAAST Team, comprising the authors of this manuscript (SM, RBrown, JS, SB, RBurn, & SO), was responsible for delivering the *Fundamentals of FASD* training and the evaluation. Furthermore, the FAAST Team receive funding from the Scottish Government to provide related training and consultancy, including the ongoing delivery of the *Fundamentals of FASD* training.

## Supporting information


Appendix S1

## Data Availability

The data that support the findings of this study are available from the corresponding author upon reasonable request.
