# Supplementary material for: The fundamentals of fetal alcohol spectrum disorder: Evaluation of an awareness‐raising webinar
Source: Alcohol Clin Exp Res (Hoboken). 2026 Feb 19;50(2):e70251. doi: 10.1111/acer.70251 (PMC12920269; doi:10.1111/acer.70251)
Supplement: Supplementary file 1 — Appendix S1 [file ACER-50-0-s001.docx]

# Supplementary Materials 1: Questionnaires

## Attitudes Towards Health Advice (T1 – T4)

The next section asks you about your beliefs and thoughts about drinking during pregnancy. We are aware that everyone has different opinions and beliefs; and health guidance on this topic has changed rapidly and still differs between countries. Please be open and honest and remember that this is a safe space. Please tell us how you truly feel about the following statements.

Please indicate how strongly you endorse each of the following health advice statements that are directed towards women of childbearing age

|  | Strongly disagree (1) | Somewhat disagree (2) | Neither agree nor disagree (3) | Somewhat agree (4) | Strongly agree (5) |
| --- | --- | --- | --- | --- | --- |
| There is no safe level of alcohol use during pregnancy |  |  |  |  |  |
| Women should avoid alcohol when trying to become pregnant |  |  |  |  |  |
| Women should avoid alcohol when they are pregnant |  |  |  |  |  |

## Attitudes Towards Prenatal Alcohol Exposure (PAE) (T1 – T4)

*(Adapted from (Peadon et al., 2010; Peadon et al., 2011)*)

The next section asks you about your beliefs and thoughts about drinking during pregnancy. We are aware that everyone has different opinions and beliefs and there are no right or wrong answers; health guidance on this topic has changed rapidly and still differs between countries. Please be open and honest and remember that this is a safe space.

Please tell us how your level of agreement with each of the following statements

|  | Strongly Disagree (1) | Disagree (2) | Neither agree nor disagree (3) | Agree (4) | Strongly Agree (5) |
| --- | --- | --- | --- | --- | --- |
| Health Professionals should advise women who are pregnant or who are thinking of becoming pregnant to give up drinking alcohol |  |  |  |  |  |
| Health professionals should ask pregnant women about how much and how often they drink alcohol |  |  |  |  |  |
| Members of the general public are concerned about women drinking alcohol during pregnancy |  |  |  |  |  |
| Information should be readily available to women about the effect that drinking alcohol may have on the unborn child |  |  |  |  |  |
| Women are aware of the effects that drinking alcohol during pregnancy can have on the unborn child |  |  |  |  |  |
| Drinking alcohol during pregnancy can lead to life-long disabilities in a child |  |  |  |  |  |
| The more alcohol a pregnant women drinks, the more likely that the unborn child will be affected |  |  |  |  |  |
| Drinking alcohol during pregnancy can affect the unborn child |  |  |  |  |  |
| It is ok for pregnant women to become intoxicated |  |  |  |  |  |
| It is ok for pregnant women to drink three or four units of alcohol in one day |  |  |  |  |  |
| Pregnant women should drink less than seven units of alcohol each week |  |  |  |  |  |
| Pregnant women should not drink alcohol |  |  |  |  |  |

## Attitudes Towards FASD (T1 – T4)

*(Adapted from Passmore et al. (2018), questions edited by the research team are highlighted in green, and new questions are highlighted in yellow)*

The next section asks you about your beliefs and thoughts about Fetal Alcohol Spectrum Disorder (FASD). We are aware that everyone has different opinions and beliefs and there are no right or wrong answers; health guidance on this topic has changed rapidly and still differs between countries. Please be open and honest and remember that this is a safe space.

Please read each statement carefully and rate how strongly you agree with each of the following statements.

|  | Strongly disagree (1) | Disagree (2) | Neither agree nor disagree (3) | Agree (4) | Strongly agree (5) |
| --- | --- | --- | --- | --- | --- |
| I am familiar with how alcohol use during pregnancy can affect fetal development |  |  |  |  |  |
| I am familiar with the difficulties people with FASD can experience |  |  |  |  |  |
| I am NOT familiar with the cause of FASD |  |  |  |  |  |
| I am familiar with how FASD can affect people's lives |  |  |  |  |  |
| FASD can be diagnosed at any age |  |  |  |  |  |
| People with FASD have permanent brain damage |  |  |  |  |  |
| Alcohol's negative effect on fetal development has been proven |  |  |  |  |  |
| Most birth mothers who drink when pregnant know it can harm the baby |  |  |  |  |  |
| All people with FASD have particular facial characteristics |  |  |  |  |  |
| People can grow out of FASD |  |  |  |  |  |
| FASD occurs primarily in financially disadvantaged families |  |  |  |  |  |
| Diagnosis of FASD would NOT improve outcomes for those affected by FASD |  |  |  |  |  |
| **The benefits of a diagnosis of FASD do not outweigh the harm it can cause to families** |  |  |  |  |  |
| **The emphasis on FASD is stigmatising to women** |  |  |  |  |  |
| **FASD is relevant to my work** |  |  |  |  |  |
| FASD is only relevant to people aged under 18 years |  |  |  |  |  |

## FASD Knowledge (T1 – T4)

The following questions are intended to measure your knowledge about alcohol use during pregnancy, and Fetal Alcohol Spectrum Disorders. Please answer each of the following questions to the best of your ability. If you don't know the answers to any of the questions, please take your best guess from the options presented.

Very few people are likely to get many of these questions before attending our training, we wouldn’t expect you to! We would like to gauge your current knowledge though so that we can quantify how effective our training is and see how it might be improved

[Answers marked as correct for calculating the Knowledge Score are highlighted in green]

What proportion of pregnant women report drinking alcohol at any time during their pregnancy in the UK?

About 10%

About 20%

About 30%

About 40%

What are some of the factors that are associated with an increased risk of alcohol consumption during pregnancy? (Check all that apply)

Experiences of trauma or interpersonal violence (IPV)

Low maternal education

Higher Socio-Economic Status (SES)

Alcohol consumption by the partner or social circle

True or false, alcohol passes freely through the placenta?

True

False

True or false, alcohol can cause damage at *any* stage of pregnancy?

True

False

What is Fetal Alcohol Spectrum Disorder?

The spectrum of physical, emotional, and developmental delays that can affect an individual throughout their lifetime as a result of changes to the brain structure and function following prenatal exposure to alcohol

The short-term physical symptoms of alcohol withdrawal that babies born to mothers who used alcohol during pregnancy experience

Impairments in cognitive development that occur when a baby is exposed to alcohol post-birth, either directly or through breast milk

An increase in alcohol consumption that accompanies post-natal depression

True or false, FASD is found across the socio-economic status spectrum?

True

False

Research with birth mothers of children with FASD has found birth mothers experience high rates of which of the following?

Experiences of abuse or neglect as a child themselves

Symptoms of Post-Traumatic Stress Disorder (PTSD)

Difficulty reducing alcohol consumption as they were in an abusive relationship

All of the above

What are some of the factors that can influence the presentation of FASD? (Check all that apply)

The amount of alcohol consumed

The gestational timing of exposure

Maternal nutrition

Pattern of consumption (i.e., binge drinking)

The price of the alcohol consumed

What is the estimated prevalence of FASD in Scotland?

Less than 1%

Between 1 and 3%

Between 3 and 5%

More than 5%

What proportion of individuals with FASD display the sentinel facial features (i.e., smooth philtrum, thin upper lip, short palpebral fissures [the horizontal opening of the eye])?

100%

50%

10%

1%

What are some of the benefits of a diagnosis of FASD for the individuals and their families?

Help ensure that individuals with FASD obtain more suitable, FASD-specific care and intervention

Can reduce FASD risk for future pregnancies

Helps families and individuals to understand the nature of the difficulties and respond appropriately

All of the above

What are some of the reasons that individuals with FASD may go undiagnosed or receive an incorrect diagnosis? (Check all that apply)

Lack of facial features

Questions about alcohol use in pregnancy not asked

Need for further training among health professionals

The behavioural presentation may be similar to other neurodevelopmental impairments including ADHD and Autism Spectrum Disorders

True or false, the early and accurate diagnosis of FASD reduces the risk of negative long-terms outcomes?

True

False

FASD may be over-represented in which of the following populations? (Check all that apply)

Care experienced / looked-after children

Criminal justice system

Homelessness

Individuals with substance use issues

## Additional Questions at T2

The following questions ask about your perceptions of the *Fundamental of Fetal Alcohol Spectrum Disorders for Health and Social Care*.

The following questions ask about your perceptions of the training.

|  | Strongly Disagree (1) | Disagree (2) | Neither agree nor disagree (3) | Agree (4) | Strongly Agree (5) |
| --- | --- | --- | --- | --- | --- |
| The training kept my interest |  |  |  |  |  |
| Through the training I became aware of how I can apply what I have learned |  |  |  |  |  |
| I consider what I learnt through the training as useful for carrying out my work |  |  |  |  |  |
| I would recommend this training to others who may work with women of child bearing age, or who may work with affected individuals |  |  |  |  |  |

What can we do to improve the FASD training course?

[Open text field for responses]

## Additional Questions at T3 and T4

Since you completed the training have you supported individuals diagnosed with or suspected of having FASD, or their families?

Yes

No

If yes, please comment on whether or not the training was useful in your work.

[Open text field for responses]

## References for measures

Passmore, H. M., Mutch, R. C., Burns, S., Watkins, R., Carapetis, J., Hall, G., & Bower, C. (2018). Fetal Alcohol Spectrum Disorder (FASD): Knowledge, attitudes, experiences and practices of the Western Australian youth custodial workforce. *International Journal of Law and Psychiatry*, *59*, 44-52. https://doi.org/10.1016/j.ijlp.2018.05.008

Peadon, E., Payne, J., Henley, N., D'Antoine, H., Bartu, A., O'Leary, C., Bower, C., & Elliott, E. J. (2010). Women's knowledge and attitudes regarding alcohol consumption in pregnancy: a national survey. *BMC Public Health*, *10*(1), 510. https://doi.org/10.1186/1471-2458-10-510

Peadon, E., Payne, J., Henley, N., D'Antoine, H., Bartu, A., O'Leary, C., Bower, C., & Elliott, E. J. (2011). Attitudes and behaviour predict women's intention to drink alcohol during pregnancy: the challenge for health professionals. *BMC Public Health*, *11*(1), 584. https://doi.org/10.1186/1471-2458-11-584

# Supplementary materials 2: Normal Q-Q Plots of Residuals


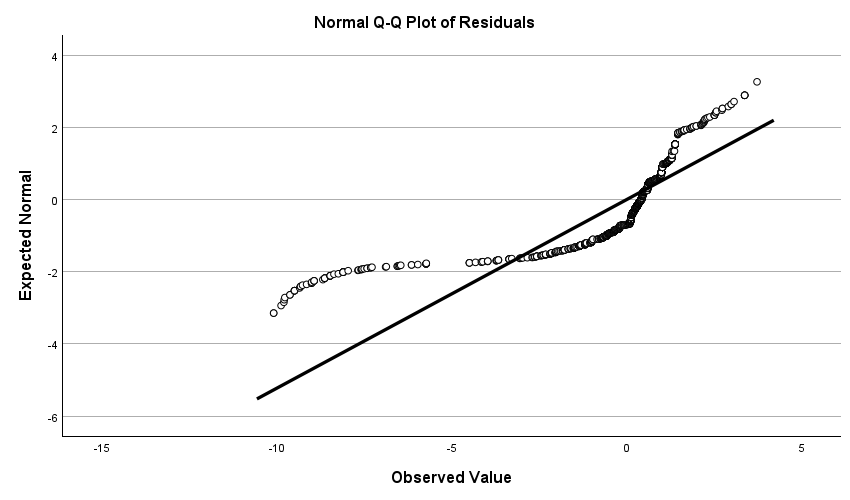


**Supplemental Figure 1.** Distribution of residuals for Health Advice Attitudes total scores.


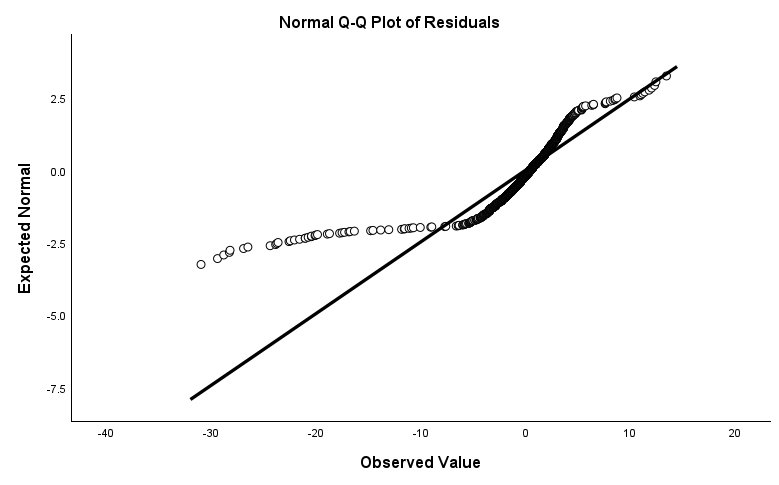


**Supplemental Figure 2.** Distribution of residuals for Prenatal Alcohol Exposure (PAE) Attitudes total scores.


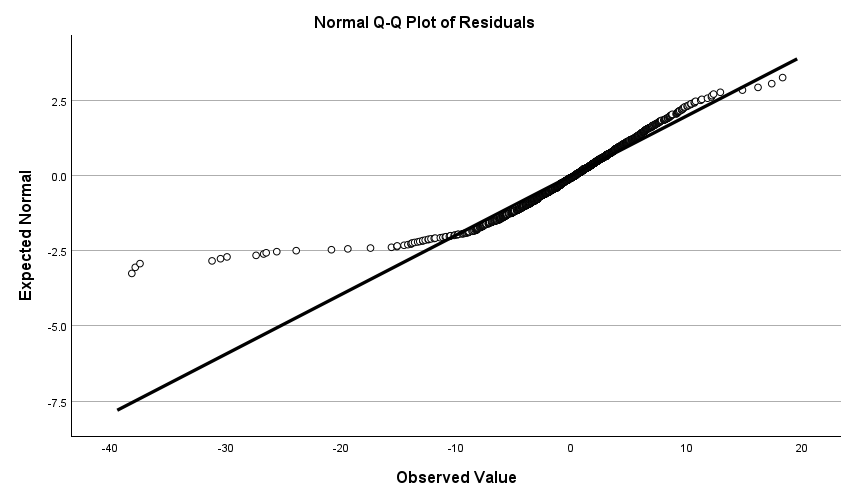


**Supplemental Figure 3.** Q-Q plot of residuals for FASD Attitudes total scores.


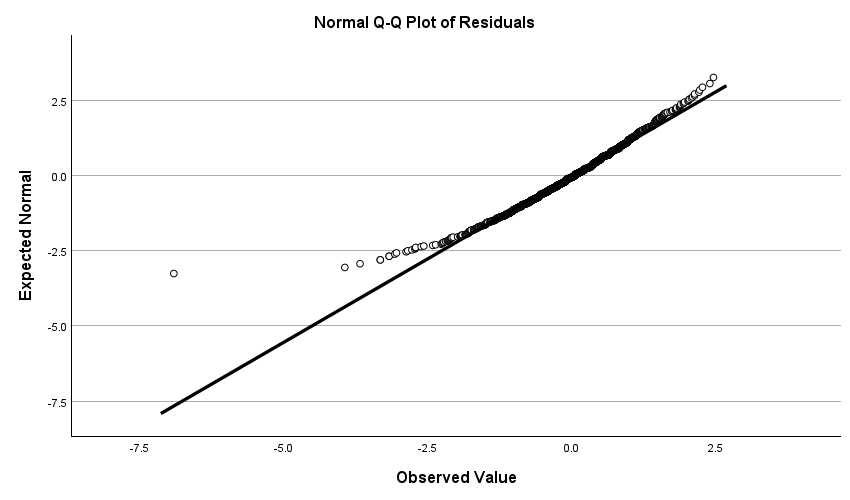


**Supplemental Figure 4.** Q-Q plot of residuals for FASD Knowledge total scores.
